# Supplementary material for: Storage and export of microbial biomass across the western Greenland Ice Sheet
Source: Nat Commun. 2021 Jun 25;12:3960. doi: 10.1038/s41467-021-24040-9 (PMC8233322; doi:10.1038/s41467-021-24040-9)
Supplement: Supplementary file 1 — Supplementary Information [file 41467_2021_24040_MOESM1_ESM.pdf]

Supplementary Information for:

Nature Communications

Storage and export of microbial biomass across the western Greenland Ice Sheet

TDL Irvine-Fynn[1]*, A Edwards[2], IT Stevens[1,3,4], AC Mitchell[1], P Bunting[1], JE Box[5], KA Cameron[2,5,6], JM Cook[2,4,7], K Naegeli[1,8], SME Rassner[2], JC Ryan[9], M Stibal[10], CJ Williamson[11], A Hubbard[12,13]

1. Department of Geography and Earth Sciences, Aberystwyth University, Aberystwyth, SY23 3DB, UK;

2. Institute of Biological Environmental and Rural Sciences, Aberystwyth University, Aberystwyth, SY23 3DA, UK;

3. School of Geography, Politics and Sociology, Newcastle University, Newcastle-upon-Tyne, NE1 7RU, UK;

4. Department of Environmental Science, Aarhus University, Frederiksborgvej, DK-4000 Roskilde, Denmark;

5. Department of Glaciology and Climate, Geological Survey of Denmark and Greenland, Copenhagen, Denmark;

6. School of Geographical & Earth Sciences, University of Glasgow, Glasgow, G12 8QQ. UK;

7. Department of Geography, University of Sheffield, Sheffield, S10 2TN, UK;

8. Institute of Geography and Oeschger Center for Climate Change Research, University of Bern, Bern, CH-3012, Switzerland;

9. Institute at Brown for Environment and Society, Brown University, Providence, RI, 02912, USA;

10. Department of Ecology, Faculty of Science, Charles University, 12844, Prague, Czechia;

11. Bristol Glaciology Centre, School of Geographical Sciences, University of Bristol, Bristol, BS8 1SS, UK;

12. Centre for Gas Hydrate, Environment and Climate, Department of Geosciences, UiT - The Arctic University of Norway, Tromsø, N-9037, Norway.

13. Kvantum Institute, University of Oulu, Oulu, Finland.

Contents of this file:

Supplementary Methods S1 to S6
Supplementary Figures 1 and 2
Supplementary Table 1
Supplementary Equations 1 to 3
Supplementary References S1-S24

Introduction

This Supplementary Information documentation includes additional descriptions of the ice surface hydro-meteorological assessments employed in our study, the flow cytometric enumeration protocols and gating procedures employed in our flow cytometric analyses, the platform employed to acquire digital imagery of the ice surface in western Greenland, and a detailed explanation of our upscaling approach to estimate microbial cell and carbon fluxes over a wider region of the Greenland Ice Sheet.

Supplementary Method S1: Derivation of ice surface aerodynamic roughness lengths (z_0) and hydraulic conductivity (K)

The aerodynamic roughness length (z_0) of a glacier's surface imparts a control on the turbulent heat transfer from the atmosphere to the ice. Therefore, for estimates of glacier surface melt rates, many numerical models require z_0 as a parameter. Physically plausible parameterizations of z_0, based on the geometry of glacier surface topography (or roughness) and associated effective drag for turbulent flow, have been effective[S1-3]. These traditional microtopographic methods typically rely on measurements of surface elevation along a transect of length *L*, perpendicular to dominant wind direction. The linear set of surface elevation measurements are detrended and broken into sections protruding above the mean transect elevation value. These sections represent obstacles to air flow, with a frequency (*f*) defined as the number of continuous groups of positive elevation deviations above the mean elevation over length L. The method[S1-2] estimates the resistance to air flow based on the average obstacle height ($h*$) and the vertical silhouette area (*s*) and specific area measured over the horizontal plane (*S*) of the average obstacle over the transect. Because *s* and *S* are both defined by the ratio of *L* : *f* [ref.S1], the parameter z_0 can be derived as:

$$z_0 \approx \frac{1}{2} h^* \left(\frac{s}{S}\right) = {\sigma_z}^2 f / L \qquad \text{[Supp. Eq.1]}$$

where, as an approximation, the mean obstacle height ($h*$) is estimated as twice the standard deviation of elevation (σ_z) for the detrended transect. For transect lengths between 3 and 15 m, the estimate of z_0 is independent of length[S3].

Supplementary Method S2: Derivation of near-surface weathering crust hydraulic conductivity (K)

Near-surface hydraulic conductivity is commonly assessed using piezometer tests that, following a disturbance to the water level, quantify the hydrological recovery of a shallow auger hole[S4] (Amoozegar and Warwick, 1986). In the ice surface environment, positive disturbance through introduction of water to an auger hole would artificially raise the local water table. Recognising the bulk density gradient in the weathering crust, such a disturbance would likely induce water flow through higher porosity ice and an over-estimate of hydraulic conductivity. Therefore, a negative disturbance in which water is evacuated and hydraulic recovery monitored (the bail-recharge method) was considered more appropriate for this study.

We utilized a Kovacs ice auger to drill shallow holes of up to 36 cm depth in the weathering crust, and a biOrb™ syphon was employed to evacuate (bail) water from the hole. The bailed auger holes were immediately instrumented with bespoke electronic capacitance piezometers, recording the rising water level (recharge) at 2 s intervals with a Track-It USB data-logger (Monarch Instruments, USA). Our piezometers function by recording the voltage derived from the frequency of an electrical current applied across the auger hole, within the piezometer housing, which scales in proportion to capacitance: output voltage increases as the water level rises and capacitance is reduced.

Water recharge curves, plotted as water volume against time, were characterised by three stages. The first, rapid quasi-linear recharge stage, relates to pressure driven water flow into the auger hole (Stage 1). The second, non-linear stage (Stage 2), reflects the flow of water through an undisturbed weathering crust. The final, linear stage with a gradient of zero is the point at which the auger hole water level equilibrates with the weathering crust's water table (Stage 3). From these curves,

hydraulic conductivity (K) can be approximated empirically following the solution proposed by Bouwer and Rice[S5] where length scales are given in centimetres:

$$K \approx \frac{Q_r\left(\frac{1.1}{\ln\left(d_r/r_h\right)} + \frac{A+B\cdot\ln\left[\left(D_{wc}-d_r\right)/r_h\right]}{\left(d_r/r_h\right)}\right)}{2\pi d_r D_{wt}} \qquad \text{[Supp. Eq.2]}$$

where Q_r represents the recharge water flow rate ($cm^3\ s^{-1}$), defined by the auger hole geometry and rate of water column recovery measured by the piezometer and derived from recharge curve Stage 2; d_r is the depth of the auger hole through which water enters, as defined by Stage 3, and r_h is auger hole radius (2.5 cm). D_{wc} is the depth between the water table and the impermeable base of the weathering crust: for this study, we set D_{wc} = 40 cm, and such that $[(D_{wc} - d_r) / r_h] \leq 6$ [ref.S5]. A and B defined here as 2 and 0.25, respectively, are dimensionless constants related to the ratio of h_r:r_h. The final variable, D_{wt}, is the depth between the observed water surface and equilibrium water table, approximated to be immediately below the static water table such that $D_{wt} > 0$. Numerical simulations accounting for error arising from measurement precision and user determination of recharge stages revealed a mean uncertainty in K of 4.8%[S6]. Recharge curves in instances where either water level failed to recover following disturbance, or Stages 2 and/or 3 were indistinguishable were defined as 'incomplete' and reliable derivation of K was impracticable.

Supplementary Method S3: Flow cytometry protocol for the enumeration of microbial cells in supraglacial meltwaters

Flow cytometry (typically abbreviated to FCM, as here) has become a well-established and valued tool for the study of microbial communities in aquatic ecosystems[S7] and has been employed successfully in glaciological studies[S8-S10]. Such analyses can enumerate total particle loads, provide estimates on microbial abundance and size distributions, and combined with specific molecular or biological probes and/or autofluorescence is ideal for the detection of individual cell types against the backdrop of organic and inorganic particulates. Here, each sample was divided into two 1 mL

aliquots, one of which was stained using SYBR Gold (1 × final concentration) whilst the other remained unstained, acting as a negative control. Both aliquots were stored in the dark at 20 °C for 20 to 240 minutes prior to enumeration. The FCM enumerations were undertaken using a Sony SH-800EC cell sorter, configured with 488 nm forward and back scatter detectors (FSC and BSC, respectively) and six fluorescence channels, including fluorescein isothiocyanate (FITC: 525 ± 30 nm). The instrument was aligned and calibrated daily using 8-peak reference beads (Sony Biotechnology, Japan). An instrumental trigger threshold was established in the FSC-Area (FSC-A) channel using 1 µm size-calibration beads (Molecular Probes, USA) suspended in Ultrapure Water (Elga), ensuring that micron-scale particles were detected. The threshold and gain of the trigger channel was set at the lowest possible point at which no noise was observed whilst ensuring the detection of the 1 µm beads. A log-log BSC-A against fluorescence channel 2 area (FL2: FITC-A) plot was used to define the bacterial gate, via the comparison of unstained (Supplementary Fig. 1a) and stained (Supplementary Fig. 1b) sample pairs. Size gates were defined from a non-fluorescent size calibration kit (Molecular Probes, USA) enabling discrimination of microbial cells into seven size categories from the FSC channel, and bacterial size distribution was measured using these gates, as per the manufacturer's instructions (Supplementary Fig. 1c). Gates were kept consistent throughout FCM sample runs.

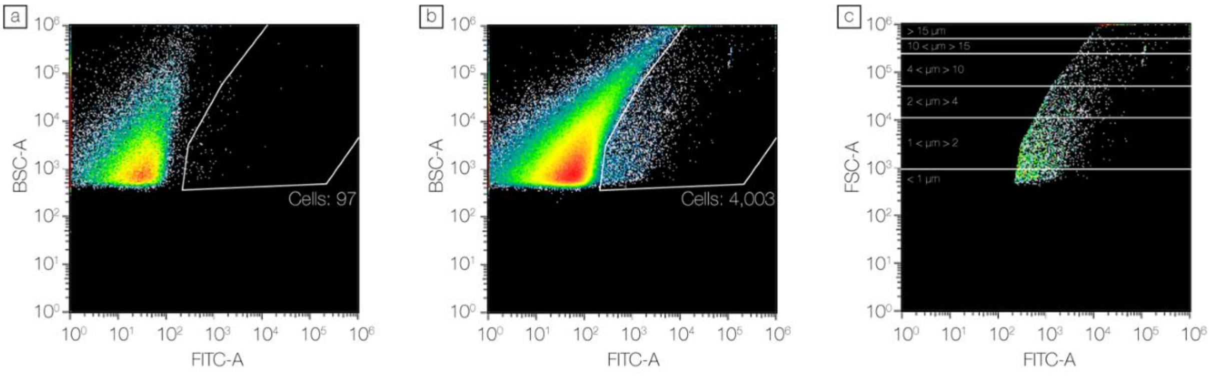

Supplementary Fig. 1: Illustration of the gating protocol used to enumerate microbe abundance and size fractions with example unstained sample (**a**), the same sample with SYBR Gold stain (**b**) and size fractions defined by the FSC-A (**c**).

Supplementary Method S4: Image acquisition using an Unmanned Aerial Vehicle (UAV)

The fixed-wing UAV used in this study was identical to those described in detailed by Ryan et al.[S5-7]. The UAV platform comprised a 2.1 m wingspan airframe, a 10 Ah 16.8 V LiPo battery, an Arduino autopilot module, and included a downward-facing Sony NEX-5N digital camera, totalling a platform weight of ~ 4 kg. The autonomous control system of the Arduino navigation and flight computer is updated in real-time. A horizontal displacement threshold was used to automatically trigger the camera shutter. The camera has a 16 mm fixed-focus lens, which yields a field-of-view of 53.1° by 73.7°, and was pre-set for a fixed exposure of 1/1000 s, ISO-100 and F-stop of 8. At an elevation of 350 m above the surface, the imaging footprint was approximately 525 x 350 m, with a pixel equating to a horizontal ground sampling resolution of 0.11 m. The digital RGB images, recorded in RAW format, were georeferenced using the latitude, longitude, altitude and attitude data recorded by the onboard flight controller. Pre-planned flight lines over the ice sheet, were uploaded to the UAV using the ArduPilot Mission Planner ground station software (http://ardupilot.org/planner/) and the image acquisition sortie was conducted on 8 August, 2014.

Supplementary Method S5: Derivation of a seasonal export of cellular carbon from the weathering crust interfluve

The porous weathering crust develops through subsurface melt arising from shortwave radiation penetration and heat transfer by percolating meltwater. However, to date, this process remains poorly constrained outside Antarctica[S14]. Therefore, as a first order estimate following Muller and Keeler[S15], we simply assumed that the weathering crust in western Greenland deepens from the initial exposure of bare-ice in early summer when short-wave radiation is elevated; in the later summer, as the shortwave radiation is reduced relative to sensible heat fluxes, the weathering crust thins and declines.

To determine the duration bare ice exposure, we used the data presented by Ryan et al.[S16] that used the MODIS MOD09GA and MOD10A1 products to define a bare ice exposure frequency over the 90-day summer season (JJA) centred on 15 July (DOY196). From these data, we extracted the mean bare ice duration (b_i) for 795 moulin terminating catchments in the low gradient, moderately crevasse-free ablation area region ranging from 400 m to 2000 m a.s.l. as delineated by Yang and Smith[S17]. The western Greenland region reported here covers a total area of 13847 km^2, with a mean internally drained catchment area of 17.4 km^2. Our ~0.5 km^2 S6 study catchment lay within a larger 43.8 km^2 moulin-terminating catchment that, in 2014, exhibited an areal mean bare ice duration of 68 days.

In the absence of any data pertaining to weathering crust development, we restrict its thickness to 0.4 m with a maximum hydraulically active depth (W_{max}) of 0.29 m, as defined by our recharge experiments and observations of the near-surface water table. To model the seasonal evolution of the hydraulically active layer we used a cosine function. To define this function, we employed the 68-day bare ice duration for the wider S6 study catchment, and our observation period from DOY 204-211 throughout which W_{max} was assumed constant (Supplementary Fig. 2). This approach yielded a 30-day period with the hydraulically active layer assumed to be W_{max}, and 19-day phases of weathering crust growth and decay. For bare ice durations less than 38 days, we assumed a linear increase in the depth of the weathering crust's hydraulically active layer based on b_i.

To define the water discharged from the supraglacial interfluve area over the bare-ice period (b_i), we integrate the depth function over t days, and multiply by the weathering crust effective porosity (ϕ = 0.22), our derivation of throughflow velocity (v_t) and the surface fraction (S_f) we expect to represent ice adjacent to a stream:

$$Q_{wc} = \begin{cases} S_f \phi v_t \left(\int_{t=0}^{b_i} \frac{b_i W_{max}}{2 b_p} \left(\cos\left(\pi \left(\frac{2t}{b_i} - 1 \right) \right) + 1 \right) \mathrm{d}t \right) & : b_i \leq b_p \\ S_f \phi v_t \left(\int_{t=0}^{b_p} \frac{W_{max}}{2} \left(\cos\left(\pi \left(\frac{2t}{b_p} - 1 \right) \right) + 1 \right) \mathrm{d}t \right) + W_{max}(b_i - b_p) & : b_i > b_p \end{cases} \quad \text{[Supp. Eq.3]}$$

Based on our study catchment, the interfluve stream bank area across the region was kept constant at S_f = 14%, and the bare ice duration that sees the hydraulically active weathering crust layer peak but not remain at W_{max} (b_p) was 38 days.

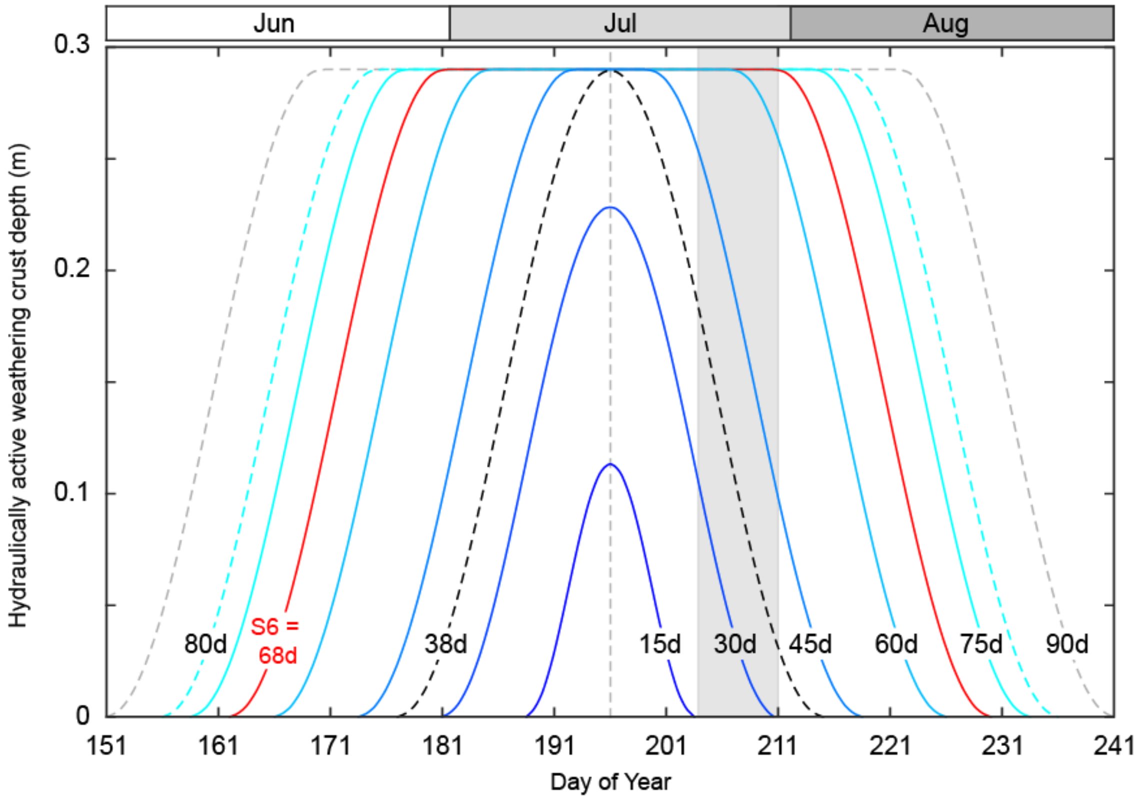

Supplementary Fig. 2: Illustration of the evolving hydraulically active weathering crust depth for varied bare ice durations at 15-day intervals used to simulate the seasonal growth and decay of the porous surface ice layer. The 90-day summer (JJA) season is centred on 15 July (DOY 196). The 2014 field campaign, from DOY204-211, is shown in grey with the hydraulically active weathering crust depth as derived for the 68-day bare-ice duration observed in the region of our study catchment. The maximum mean bare-ice duration observed in the catchments in the western sector of the ice sheet was 80 days.

During 2014, the average areal mean bare ice duration for catchments over the western Greenland region was 42.8 d (ranging from 0.1 d to 75.7 d). In contrast, for the low melt year of 2006, this value was reduced to 22.8 d (ranging from 0 to 62.2 d), while for the high melt observed in 2012 durations increased to 60.4 d (from 15.9 to 80.4 d). From the ranges of b_i, the discharge (Q_{wc}) for each catchment across the was calculated, converted to a flux of microbial cells and then presented as

biomass of carbon (see Supplementary Table 1). Our approach here provides recognition of the latitudinal and elevational variation in weathering crust development, and associated drainage evolution. The derivation of an active stream network from remote sensing data will be conditioned by the resolution of the imagery employed, as well as the seasonal evolution of the surface drainage density; these variables are beyond the scope of this study. However, it is clear more detailed modelling of both weathering crust development[S14] in the context of Greenland, and determination of seasonal evolution in drainage density as well as the temporal and spatial variation in both the intrinsic and extrinsic controlling factors influencing the hydraulic conductivity of the weathering crust[S6] are required to further refine our estimates of carbon fluxes at wider regional scales.

Supplementary Method S6: Numerical analyses and figure design

Commercially available software utilised in the numerical analyses, and development of illustrations presented in the manuscript and Supplementary Information included Agisoft PhotoScan Pro, ESRI ArcGIS v10.5, MS Excel 2016, Matlab2014b, Sony Cell Sorter v.2.1.3, and Illustrator CS3.

Supplementary Table 1: Seasonal estimates of microbe abundance and associated mass of cellular carbon (in kg C) exported from the weathering crust to the supraglacial stream at the S6 study site in 2014, and to the subglacial environment in the western sector of the ice sheet based on moulin terminating catchments extending over an area of 1.38 $\times 10^{-4}$ km^2. Results are also shown for low (2006) and high (2012) melt years. Biomass estimates are made for the ≤ 15 µm size classes and for all classes including large (>15 µm) algae, using low[S18,S19] and high[S20,S21] cell constants, allometric[S22] and constant[S23,S24] ratios.

Variable / Location		S6 study catchment 2014			Western ice sheet region 2014			Western ice sheet region 2006			Western ice sheet region 2012		
V_t	Biomass calculation	Cells	≤15 µm	All	Cells	≤15 µm	All	Cells	≤15 µm	All	Cells	≤15 µm	All
0.03	Constant (11+153 fg)	1.37×10^{14}	1.9×10^{-3}	3.2×10^{-3}	1.81×10^{18}	25.2	42.5	7.20×10^{17}	10.0	16.9	3.08×10^{18}	42.8	72.4
	Constant (20+260 fg)		3.3×10^{-3}	5.6×10^{-3}		44.9	74.5		17.9	29.6		76.4	127
	Allometric		0.29	1.2		3.8×10^{3}	1.6×10^{4}		1.5×10^{3}	6.1×10^{3}		6.5×10^{3}	2.6×10^{4}
	Constant ratio		0.35	1.3		4.6×10^{3}	1.7×10^{4}		1.8×10^{3}	6.8×10^{3}		7.8×10^{3}	2.9×10^{4}
0.13	Constant (11 + 153)	6.24×10^{14}	2.3×10^{-2}	2.4×10^{-2}	8.27×10^{18}	301	319	3.29×10^{18}	119	126	1.41×10^{19}	512	542
	Constant (20 + 260)		3.9×10^{-2}	6.9×10^{-2}		520	918		207	365		885	1.6×10^{3}
	Allometric		9.2	21.1		1.2×10^{5}	2.8×10^{5}		4.8×10^{4}	1.1×10^{5}		2.1×10^{5}	4.8×10^{5}
	Constant ratio		10.2	23.0		1.3×10^{3}	3.0×10^{5}		5.4×10^{4}	1.2×10^{5}		2.3×10^{5}	5.2×10^{5}

Supplementary References:

S1 Lettau, H. H. Note on aerodynamic roughness-parameter estimation on the basis of roughness-element description, *Journal of Applied Meteorology*, **8**, 828– 832 (1969).

S2 Munro, D. S. Surface roughness and bulk heat transfer on a glacier: Comparison with eddy correlation, *Journal of Glaciology* **35**, 343-348 (1989).

S3 Brock, B. W., Willis, I. C., and Sharp, M. J. Measurement and parameterization of aerodynamic roughness length variations at Haut Glacier d'Arolla, Switzerland, *Journal of Glaciology* **52**, 281-297 (2006).

S4 Amoozegar A., Warrick A.W. In Methods of Soil Analysis: Part 1. Physical and Mineralogical Methods (ed A Klute), 735-770 (Soil Science Society of America, 1986).

S5 Bouwer, H., Rice, R. C. A slug test determining hydraulic conductivity of unconfined aquifers with completely or partially penetrating wells. *Water Resources Research* **12**, 423-428 (1976).

S6 Stevens, I. T. et al. Near-surface hydraulic conductivity of northern hemisphere glaciers. Hydrological Processes **32**, 850-865 (2018).

S7 Wang, Y., Hammes, F., De Roy, K., Verstraete, W. and Boon, N. Past, present and future applications of flow cytometry in aquatic microbiology. *Trends in Biotechnology* **28**, 416-424 (2010).

S8 Irvine-Fynn, T. D. L. et al. Microbial cell budgets of an Arctic glacier surface quantified using flow cytometry. *Environmental Microbiology* **14**, 2998-3012 (2012).

S9 Stibal, M. et al. Microbial abundance in surface ice on the Greenland Ice Sheet. *Frontiers in Microbiology* **6**, 00225 (2015).

S10 Santibanez, P. A., McConnell, J. R. & Priscu, J. C. A flow cytometric method to measure prokaryotic records in ice cores: an example from the West Antarctic Ice Sheet Divide drilling site. *Journal of Glaciology* **62**, 655-673 (2016).

S11 Ryan, J. C. et al. How robust are in situ observations for validating satellite-derived albedo over the dark zone of the Greenland Ice Sheet? *Geophysical Research Letters* **44**, 6218-6225 (2017)

S12 Ryan, J. C. et al. Dark zone of the Greenland Ice Sheet controlled by distributed biologically-active impurities. *Nature Communications* **9**, 1065 (2018).

S13 Ryan, J. C. et al. Derivation of high spatial resolution albedo from UAV digital imagery: Application over the Greenland Ice Sheet. *Frontiers in Earth Science* **5**, 00040 (2017).

S14 Hoffman, M. J., Fountain, A. G. & Liston, G. E. Near-surface internal melting: a substantial mass loss on Antarctic Dry Valley glaciers. *Journal of Glaciology* **60**, 361-374 (2014).

S15 Müller, F. & Keeler, C. M. Errors in short-term ablation measurements on melting ice surfaces. *Journal of Glaciology* **8**, 91-105 (1969).

S16 Ryan, J. C. et al. Greenland Ice Sheet surface melt amplified by snowline migration and bare ice exposure. *Science Advances* **5**, eaav3738 (2019).

S17 Yang, K. & Smith, L. C. Internally drained catchments dominate supraglacial hydrology of the southwest Greenland Ice Sheet. *Journal of Geophysical Research – Earth Surface* **121**, 1891-1910 (2016).

S18 Rassner, S. M. E. et al. Can the bacterial community of a High Arctic glacier surface escape viral control? *Frontiers in Microbiology* **7**, 00956 (2016).

S19 Hamasaki, K., Satoh, F., Kikuchi, T., Toda, T. & Tagichi, S. Biomass and production of cyanobacterial in a coastal water of Sagami Bay, Japan. *Journal of Plankton Research* **21**, 1583-1591 (1999).

S20 Lee, S. & Fuhrman, J. A. Relationships between biovolume and biomass of naturally derived marine bacterioplankton. *Applied and Environmental Microbiology* **53**, 1298-1303 (1987).

S21 Montagnes, D. J. S., Berges, J. A., Harrison, P. J. & Taylor, F. J. R. Estimating carbon, nitrogen, protein, and chlorophyll-a from volume in marine-phytoplankton. *Limnology and Oceanography* **39**, 1044-1060 (1994).

S22 Norland, S. in Handbook of Methods in Aquatic Microbial Ecology (ed PF Kemp, Sherr BF, Sherr, EB, Cole, JJ), 303-307 (L Erlbaum Associates, 1993).

S23 Bratbak, G. Bacterial biovolume and biomass estimations. *Applied and Environmental Microbiology* **49**, 1488-1493 (1985).

S24 Strathmann, R. R. Estimating organic carbon content of phytoplankton from cell volume or plasma volume. *Limnology and Oceanography* **12**, 411-418 (1967).
