# Supplementary material for: Storage and export of microbial biomass across the western Greenland Ice Sheet
Source: Nat Commun. 2021 Jun 25;12:3960. doi: 10.1038/s41467-021-24040-9 (PMC8233322; doi:10.1038/s41467-021-24040-9)
Supplement: Supplementary file 3 — Reporting Summary [file 41467_2021_24040_MOESM3_ESM.pdf]

nature research

Corresponding author(s): Tristram DL Irvine-Fynn

Last updated by author(s): May 11, 2021

Reporting Summary

Nature Research wishes to improve the reproducibility of the work that we publish. This form provides structure for consistency and transparency in reporting. For further information on Nature Research policies, see our Editorial Policies and the Editorial Policy Checklist.

Statistics

For all statistical analyses, confirm that the following items are present in the figure legend, table legend, main text, or Methods section.

n/a	Confirmed	
☐	☒	The exact sample size (*n*) for each experimental group/condition, given as a discrete number and unit of measurement
☐	☒	A statement on whether measurements were taken from distinct samples or whether the same sample was measured repeatedly
☒	☐	The statistical test(s) used AND whether they are one- or two-sided<br>*Only common tests should be described solely by name; describe more complex techniques in the Methods section.*
☐	☒	A description of all covariates tested
☐	☒	A description of any assumptions or corrections, such as tests of normality and adjustment for multiple comparisons
☐	☒	A full description of the statistical parameters including central tendency (e.g. means) or other basic estimates (e.g. regression coefficient) AND variation (e.g. standard deviation) or associated estimates of uncertainty (e.g. confidence intervals)
☐	☒	For null hypothesis testing, the test statistic (e.g. *F*, *t*, *r*) with confidence intervals, effect sizes, degrees of freedom and *P* value noted<br>*Give P values as exact values whenever suitable.*
☒	☐	For Bayesian analysis, information on the choice of priors and Markov chain Monte Carlo settings
☒	☐	For hierarchical and complex designs, identification of the appropriate level for tests and full reporting of outcomes
☒	☐	Estimates of effect sizes (e.g. Cohen's *d*, Pearson's *r*), indicating how they were calculated

Our web collection on statistics for biologists contains articles on many of the points above.

Software and code

Policy information about availability of computer code

Data collection	Commercially available software and applications used for data collection: Monarch Track-It Datalogger 1.0.29; ArduPilot Mission Planner 1.3.38; propriety Sony SH800 application Sony Cell Sorter v.2.1.3
Data analysis	Commercially available software and applications used in this work: Agisoft PhotoScan Pro; ESRI ArcGIS v10.5; Matlab2014b; R; MS Excel (Office 16); Python scripts (RSGISLib) available at https://www.rsgislib.org/

For manuscripts utilizing custom algorithms or software that are central to the research but not yet described in published literature, software must be made available to editors and reviewers. We strongly encourage code deposition in a community repository (e.g. GitHub). See the Nature Research guidelines for submitting code & software for further information.

Data

Policy information about availability of data

All manuscripts must include a data availability statement. This statement should provide the following information, where applicable:

- Accession codes, unique identifiers, or web links for publicly available datasets
- A list of figures that have associated raw data
- A description of any restrictions on data availability

The datasets generated during and/or analysed in this study are available in the Zenodo repository (https://doi.org/10.5281/zenodo.4623697). Source imagery files from the UAV collected on 8 August 2014 are archived in the Pangaea repository (https://doi.org/10.1594/PANGAEA.885798) and available from the corresponding author on reasonable request. The energy balance model is available on Zenodo (https://doi.org/10.5281/zenodo.3228331). ArcticDEM data is available via https://www.pgc.umn.edu/data/arcticdem/ and RSGISLib at https://www.rsgislib.org/; the S6 weather station data is available on request from the Institute for Marine and Atmospheric Research of Utrecht University.

April 2020

Field-specific reporting

Please select the one below that is the best fit for your research. If you are not sure, read the appropriate sections before making your selection.

☐ Life sciences ☐ Behavioural & social sciences ☒ Ecological, evolutionary & environmental sciences

For a reference copy of the document with all sections, see nature.com/documents/nr-reporting-summary-flat.pdf

Ecological, evolutionary & environmental sciences study design

All studies must disclose on these points even when the disclosure is negative.

Study description	The study presents the first quantitative assessment of microbe transport through the near-surface of the Greenland Ice Sheet and the resultant cellular carbon fluxes. Applying applied standard groundwater techniques the study determines the conductivity of the near-surface ice, while flow cytometry is employed to quantify the abundance of microbes entrained in the meltwater. A first-order, kilometre-scale catchment model of microbial cell transport is developed by applying discrete observations across a high-resolution digital elevation model (DEM) derived from unmanned aerial vehicle (UAV) surveys. Analysis reveals in-situ cellular accumulation exceeds microbial export at the ice sheet surface.
Research sample	The samples reported are recharge rates from bail-recharge groundwater experiments on the surface ice of the Greenland Ice Sheet, and undefined cell abundances in the recharge meltwaters and ancillary water abstracted from trial-experiments or local cryoconite holes. Sample sites were located on 'clean' bare ice, in a quasi-randomised grid, and experiments were repeated numerous times each day over 4 days of experiments. The location of the sample sites were elected opportunistically, selecting interfluve ice areas uninterrupted by surface streams or cryoconite and with topography broadly reflective of the overarching study site. Meltwater samples were extracted following recharge experiments conducted during the course of the to determine water and microbe fluxes through the near-surface ice.
Sampling strategy	No pre-determination of sample numbers or sites were undertaken. A total of 9 experimental auger hole sites, separated by distances of ~ 9 m, were located in a quasi-random grid. Bail-recharge experiments were undertaken at these sites as frequently as possible, but determined by the variance in meltwater recharge. UAV flight was effected using Mission Planner to survey a broad area across the western region of the ice sheet's bare ice area, but was centered on and included the study catchment. Image acquisition interval was optimised for photogrammetric processing (see Ryan et al., 2015, The Cryosphere).
Data collection	Lead author conducted bail-recharge experiments using bespoke piezometers and Track-It data loggers, and abstracted meltwater samples. Third author analysed recharge data and undertook flow cytomety enumeration using a Sony SH-800EC Cell-Sorter and Sony Cell Sorter v.2.1.3 software. Fifth, ninth, eleveth and fourteenth authors were responsible for the UAV data collection (using an airframe controlled by an Arduino autopilot module imaging with a Sony NEX-5N digital camera), and its pre- and post-processing using Agisoft Photoscan Pro and ESRI's ArcGIS v.10.5. Additional meteorological data is freely available from the Institute for Marine and Atmospheric Research of Utrecht University.
Timing and spatial scale	Between July 22 and 30, 2014, experiments were conducted over a ~400 m^2 bare ice area. A total of 9 experimental auger hole sites, separated by distances of ~ 9 m, were located in a quasi-random 3x3 grid aligned perpendicular to the primary channel flowing to the northwest spread over a representative bare-ice area at sites unaffected by surface streams or cryoconite deposits. Bail-recharge measurements to derive hydraulic conductivity (K) were conducted opportunistically over 4 days within this period (July 23,25,26,29), on good-weather days; meltwater sampling was undertaken following apparent completion of auger-hole recharge. Variance in recharge rates made systematic timing of experiments across the experimental grid unfeasible throughout each day. Meltwater in each auger hole was initially evacuated and a bespoke piezometer placed to record water recharge. Piezometers were left recording until the water level in the auger hole appeared to equilibriate with the local water table at time-scales of minutes to an hour or more. Subsequently, the recharge water was sampled and a new auger hole recharge experiment was initiated to sample data at discrete times reflecting diurnal changes in surface ice melting, melt rates, and radiative energy as each experimental day progressed. Immediately following the field recharge experiments (Aug 8), while bare-ice surface conditions persisted, the UAV was flow surveying an area extending 10s of kilometres, including the study catchment of 0.5 km^2.
Data exclusions	Recharge experiments that were incomplete meant K-values were unresolvable and so excluded. Meltwater samples damaged in transit were not analysed. Following enumeration, cell abundances > 10^5 were treated as outliers and are reported as such: ten samples from sites proximate to highly localised microbial blooms and/or anomalously high particle concentrations exhibited elevated abundance, in excess of those reported by other authors for sediment-free glacier meltwaters. Image quality checks, within the Agisoft PhotoScan Pro workflow, automatically excludes low-quality units.
Reproducibility	Field-based experiments are not reproducible owing to the spatial and temporal heterogeneity of ice surface conditions. Similarly the UAV survey provides a singular snapshot of the ice surface conditions and morphology. In-depth assessment of the confidence in accuracy and precision of flow cytometric enumeration using the procedures employed had been undertaken, where replicates and constrained samples had been utilised to determine the uncertainty (<10%) in quantification of cell counts, with an RSD of 7% between triplicates.
Randomization	Randomisation is not applicable to this study, as all datasets are individual, discrete observations, which are then examined for correlation or covariant behaviour.
Blinding	No data blinding is applicable to this study, in the absence of participants, or the potential for bias arising from a quantitative analysis of discrete samples.

Did the study involve field work? ☒ Yes ☐ No

Field work, collection and transport

Field conditions	In the summer of 2014, discrete sampling was undertaken at a site located on ice sheet's western margin characterised by predominantly clear-sky conditions with a mean 2-m air temperature of 1.9°C and consistent diurnal melt variability, typically peaking at 13:00-14:00.
Location	The study site was located at 67° 04.78'N, 49° 24.08'W, 38 km from the ice sheet margin, at an elevation of approximately 1020 m a.s.l. within the well-reported Dark Zone'.
Access & import/export	Meltwater was collected using a polyethylene syringe and 30 cm polypropylene tube and decanted into a 15 mL sterile polyethylene centrifuge tube, and fixed using 50 µL glutaraldehyde (2% w/v final concentration). The preserved 10 mL samples were kept dark and cool (~ 4 °C) for up to 8 days while in the field, in insulated containers. Subsequently, transport of fixed water samples to the UK employed insulated containers carried as hold luggage with minimized transit time; all samples were subsequently fast frozen within 20 days of abstraction and stored at -80 °C until analysis.
Disturbance	Experimental procedures in an area of active ice-melt caused no disturbance.

Reporting for specific materials, systems and methods

We require information from authors about some types of materials, experimental systems and methods used in many studies. Here, indicate whether each material, system or method listed is relevant to your study. If you are not sure if a list item applies to your research, read the appropriate section before selecting a response.

Materials & experimental systems

n/a	Involved in the study	
☒	☐	Antibodies
☒	☐	Eukaryotic cell lines
☒	☐	Palaeontology and archaeology
☒	☐	Animals and other organisms
☒	☐	Human research participants
☒	☐	Clinical data
☒	☐	Dual use research of concern

Methods

n/a	Involved in the study	
☒	☐	ChIP-seq
☐	☒	Flow cytometry
☒	☐	MRI-based neuroimaging

Flow Cytometry

Plots

Confirm that:

☒ The axis labels state the marker and fluorochrome used (e.g. CD4-FITC).

☒ The axis scales are clearly visible. Include numbers along axes only for bottom left plot of group (a 'group' is an analysis of identical markers).

☒ All plots are contour plots with outliers or pseudocolor plots.

☒ A numerical value for number of cells or percentage (with statistics) is provided.

Methodology

Sample preparation	Water samples taken on site were decanted into a 15 mL sterile polyethylene centrifuge tube, and fixed using 50 µL glutaraldehyde (2% w/v final concentration). The preserved samples were kept dark and cool (~ 4 °C) for up to 8 days while in the field and in transit, and subsequently fast frozen and stored at -80 °C until analysis. Samples were thawed at ambient laboratory room temperature, gently agitated, and stained with SYBR-Gold (Molecular Probes, UK) at a final concentration of 1× and stored in the dark at 20°C for a maximum of 240 minutes prior to analysis. All experimental aliquots were vortexed for ≥ 30 seconds to ensure disaggregation of particles and even dispersal of cells throughout the suspension. Samples exhibiting 'clumping' (evident on the FSC-A and FSC-H plot) were vortexed for a second time. Enumeration was conducted using the 488 nm blue laser excitation, using the FITC-A (FL2) and FSC-A (FSC) plot. Typical flow rates used herein range from 21 µL min-1 to 63 µL min-1, with minimum time periods for analysis of 400 µL ranging from 19:02 to 4:29 minutes. An automated cleaning procedure was carried out between measurement of each experimental aliquot.
Instrument	Sony SH800EC Cell Sorter (Sony Biotechnology, Japan)
Software	Proprietary Sony operating software package for the SH800 series: Sony Cell Sorter v.2.1.3
Cell population abundance	The 73 water samples recovered from both fully and partially recharged auger holes in bare-ice show a mean microbe abundance of 2.28 ×10^4 cells mL^-1 (±1.91 ×10^4 cells mL^-1 standard deviation). Of all samples enumerated, including

outliers, abundance ranged from 2.29 ×10^4 cells mL^-1 to 1.07 ×10^6 cells mL^-1. The cell size fractions from the non-outlying samples revealed abundance proportions (and standard deviation) of: <1µm (18.5 ±5.5%); 1-2µm (49.8 ±7.0%);2-4µm (14.3 ±3.5%);4-10µm (8.7 ±4.9%);10-15µm (2.5 ±2.6%);1µm (6.3 ±3.9%).

Gating strategy

Gates to describe stained and non-stained particles were optimised for the sample water type; by comparison of stained and unstained paired samples, non-nucleic acid containing material were removed from the enumeration. The low concentrations of suspended mineral particles returned an estimated uncertainty in microbe abundance estimates of ~10% (RSD = 7%). Discrimination of cell size categories ranging from < 1 to > 15 um were estimated using a non-fluorescent Flow Cytometry Size Calibration Kit (Molecular Probes, UK) following the manufacturer's instructions, and defining 'size' on the FSC-A (FSC) axis.

☒ Tick this box to confirm that a figure exemplifying the gating strategy is provided in the Supplementary Information.
